# Decoding the role of non-coding RNAs in HNSCC radioresistance: molecular mechanisms and clinical implications

**DOI:** 10.3389/fonc.2026.1926287

**Published:** 2026-09-09

**Authors:** Feng-Xin Ge, Hui Yang, Xiang-Ze Wang, Zi-Hui Liu, Duo Shao, Hong-Xia Liu

**Affiliations:** 1 School of Stomatology, Henan University, Kaifeng, Henan, China; 2 School of Medical, Huanghe Science & Technology University, Zhengzhou, Henan, China

**Keywords:** HNSCC, lncRNAs, miRNAs, ncRNAs, radioresistance

## Abstract

Head and neck squamous cell carcinomas (HNSCC) are the sixth most commonly diagnosed cancer globally, with a stagnant five-year survival rate of approximately 50%. Risk factors include human papillomavirus (HPV) infection, smoking, alcohol consumption, and betel nut chewing, contributing to its genetic and molecular heterogeneity. Although radiotherapy is a primary treatment modality for locally advanced or inoperable HNSCC, the inherent radioresistance of tumor tissues limits its effectiveness, emphasizing the need for strategies to enhance radiosensitivity. Emerging evidence highlights the critical role of non-coding RNAs (ncRNAs), including microRNAs (miRNAs), long non-coding RNAs (lncRNAs), and circular RNAs (circRNAs), in regulating tumor radiosensitivity by modulating cell cycle, DNA damage response (DDR), cell proliferation, invasion, and migration. This review systematically examines the molecular mechanisms and clinical significance of ncRNAs in the radioresistance of HNSCC, summarizing 118 ncRNAs (from 103 articles), including 31 radioresistant miRNAs, 51 radiosensitive miRNAs, 22 radioresistant lncRNAs, 4 radiosensitive lncRNAs, 7 radioresistant circRNAs, and 3 radiosensitive circRNAs. Furthermore, this study discusses the potential roles of ncRNAs, specifically their use as predictive biomarkers for response to radiotherapy and as therapeutic targets in precision oncology. By integrating molecular insights with clinical perspectives, this review provides a valuable framework for understanding ncRNA-mediated radioresistance and proposes new strategies to overcome the challenges in improving HNSCC treatment.

## 1 Introduction

Head and neck squamous cell carcinomas (HNSCC) represent a heterogeneous group of malignancies arising from the mucosal epithelium of the oral cavity, oropharynx, larynx, and hypopharynx (1). As the sixth most commonly diagnosed malignancy globally, HNSCC accounts for over 600,000 new cases annually (2). The main risk factors for the development of HNSCC include high-risk HPV infection, smoking, alcohol consumption, and betel nut chewing. These risk factors may lead to genetic and molecular differences in tumor tissues of patients (3). Over the past few decades, researchers have developed a better understanding of the pathogenesis and progression of HNSCC, but these efforts have yielded limited benefits for clinical outcomes, as the five-year survival rate has stagnated at approximately 50% without significant improvement (4, 5).

Radiotherapy is an important treatment modality for HNSCC, especially for locally advanced or inoperable HNSCC (6). It can effectively reduce tumor volume, control local progression, and improve patient survival outcomes. However, given the inherent radioresistance of tumor tissues, not all radiotherapy treatments achieve the desired outcomes, with radioresistance being the primary cause of suboptimal outcomes (7). Therefore, there is an urgent need for approaches to overcome radioresistance in HNSCC and enhance its radiosensitivity, thereby allowing patients to derive greater benefit from radiotherapy.

Non-coding RNAs (ncRNAs), including microRNAs (miRNAs), lncRNAs, and circular RNAs, play crucial roles in gene expression regulation (8, 9). In recent years, there has been a growing focus on ncRNAs’ regulation of tumor radiosensitivity, with miRNAs drawing the most attention in this field. miRNAs, as a class of small endogenous ncRNA molecules, have become key players in elucidating the mechanisms underlying radioresistance in many malignancies, due to their central role in regulating gene expression (10). miRNAs regulate oncogenes or tumor suppressor genes by targeting their mRNAs, influencing various biological processes and behaviors of tumor cells, thereby leading to changes in tumor radioresistance (11). The biological processes mainly include cell cycle regulation and DNA repair, while biological behaviors primarily include proliferation, invasion, metastasis, migration, and apoptosis. For example, microRNA-96 (miR-96) has been shown to target phosphatase and tensin homolog deleted on chromosome ten (*PTEN)* to affect the phosphatidylinositol 3-kinase (PI3K)/protein kinase B (Akt) pathway to promote migration of HNSCC cells, thereby enhancing radioresistance in HNSCC (12). Additionally, microRNA-29a (miR-29a) is capable of suppressing ADAM metallopeptidase domain 12 (ADAM12), thereby inducing tumor apoptosis and enhancing radiosensitivity in oral squamous cell carcinoma (OSCC), a major subtype of HNSCC (13).

Compared to studies investigating the roles of miRNAs in tumor radioresistance, the number of studies focusing on lncRNAs and circRNAs in this context is relatively limited. LncRNAs indirectly promote the expression of miRNA target genes by sponging miRNAs and reducing their availability (14). For instance, the lncRNA homeobox A11 antisense RNA has been demonstrated to diminish the radiosensitivity of OSCC. This outcome is accomplished through targeting the miR-518a-3p/pyruvate dehydrogenase kinase 1 signaling axis and regulating the stemness of cancer stem cells (CSCs) in OSCC, thereby mediating the decreased radiosensitivity of the tumor (15). CircRNAs are a special class of ncRNA molecules with a closed-loop structure, which distinguishes them from conventional linear RNAs (16). CircRNAs act as “sponges” for miRNAs by binding and sequestering miRNAs, thus preventing miRNAs from binding to their respective target mRNAs (17). Due to their closed-loop structure, circRNAs are much more structurally stable than linear RNAs, allowing them to function as long-lasting and potent miRNA sponges in cells.

Within this article, we performed a systematic review to summarize the molecular mechanisms and clinical implications of ncRNAs in radioresistance associated with HNSCC. A total of 118 ncRNAs with proven molecular mechanisms or clinical significance that were included in 103 articles were analyzed. According to their functions, these ncRNAs were classified into 31 radioresistant miRNAs, 51 radiosensitive miRNAs, 22 radioresistant lncRNAs, 4 radiosensitive lncRNAs, 7 radioresistant circRNAs, and 3 radiosensitive circRNAs. Currently, most studies focus on miRNAs, while research focused on lncRNAs and circRNAs is still limited. This systematic review not only comprehensively catalogues 118 ncRNAs implicated in HNSCC radioresistance, but also systematically classifies their molecular functions within a unified framework of biological processes and cellular phenotypes. Furthermore, we explored the prognostic role of these ncRNAs in HNSCC patients after radiotherapy.

## 2 Methods

**Figure 1:**
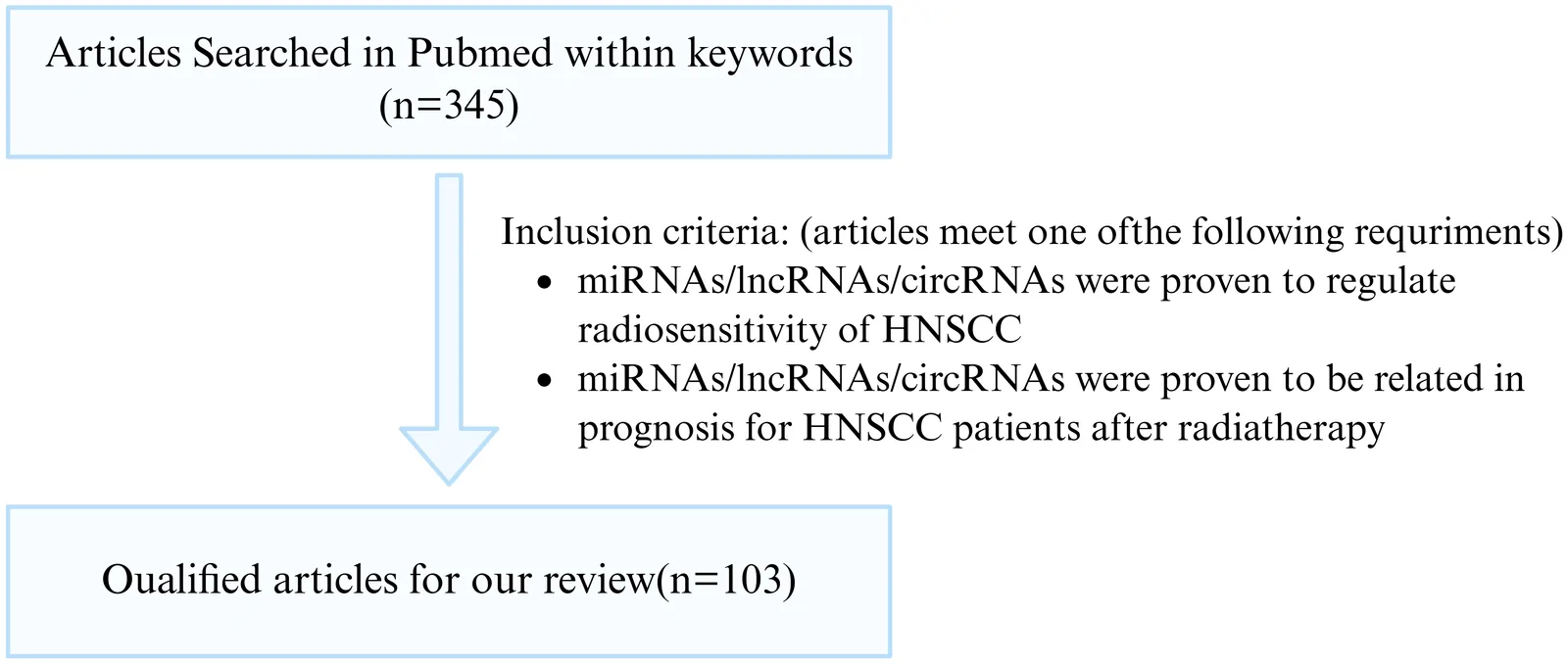
The process of articles selection. Initially, 345 articles were retrieved from PubMed using relevant keywords. Then, articles were screened according to the inclusion criteria: either miRNAs/lncRNAs/circRNAs were proven to regulate the radiosensitivity of HNSCC, or miRNAs/lncRNAs/circRNAs were proven to be associated with the prognosis of HNSCC patients after radiotherapy. Eventually, 103 articles were qualified for this review.

HNSCC comprises four major subtypes: OSCC, oropharyngeal squamous cell carcinoma, laryngeal squamous cell carcinoma, and hypopharyngeal squamous cell carcinoma (18, 19). In addition, nasopharyngeal carcinoma (NPC) is predominantly of squamous cell carcinoma origin. Also, by reviewing descriptions of cell lines in NPC-related studies, researchers could confirm whether the experimental cell lines used are of squamous cell carcinoma origin (20). Additionally, both lncRNAs and circRNAs serve as post-transcriptional regulators of miRNAs through molecular sponging, which provides the theoretical basis for exploring their synergistic regulatory roles in tumor radioresistance (21). We systematically developed and applied the following search strategy: ((lncRNA) OR (circRNA) OR (miRNA)) and ((radioresistance) OR (radiosensitivity)) and ((head and neck squamous cell carcinoma) OR (oral squamous cell carcinoma) OR (laryngeal squamous cell carcinoma) OR (oropharyngeal squamous cell carcinoma) OR (hypopharyngeal squamous cell carcinoma) OR (nasopharyngeal carcinoma)). A total of 345 articles were initially retrieved from PubMed on August 8, 2026; of these articles written in English that met at least one of the following criteria were included in this review (1): lncRNAs/circRNAs/miRNAs were shown to regulate HNSCC radiosensitivity (2); lncRNAs/circRNAs/miRNAs were shown to be associated with the prognosis of HNSCC patients after radiotherapy. Ultimately, 103 articles were included in this systematic review (**Figure 1**). The detailed inclusion and exclusion criteria for eligible articles were compiled in **Supplementary Table 1**.

## 3 Molecular mechanisms of miRNAs in radioresistance of HNSCC

### 3.1 Overall view

**Figure 2:**
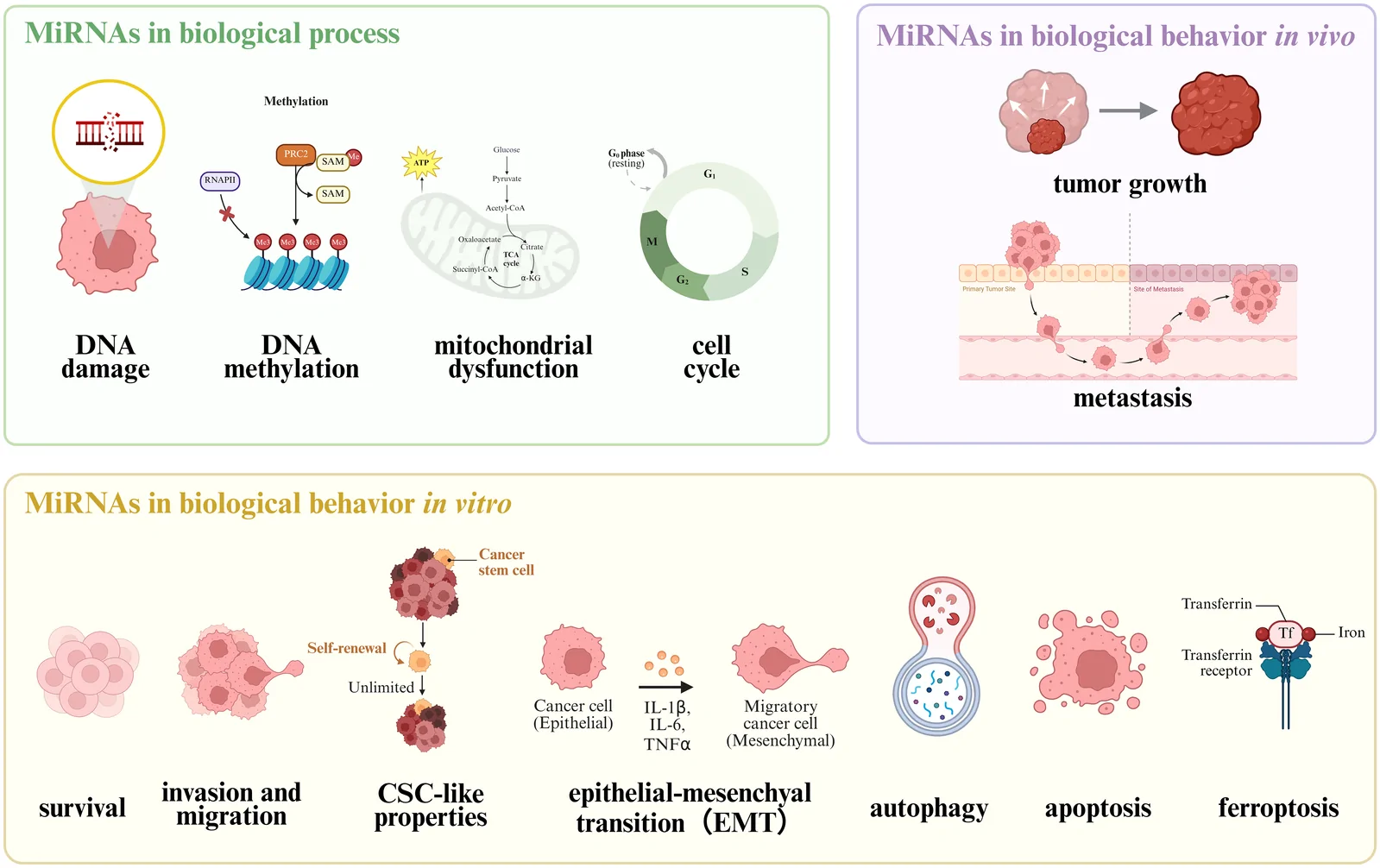
The molecular mechanism of non-coding RNAs in radioresistance of HNSCC. This figure illustrates the molecular mechanisms by which miRNAs, lncRNAs, and circRNAs mediate radioresistance in HNSCC through regulating biological processes and cellular behaviors.

miRNAs play a crucial role in the molecular mechanisms underlying radiotherapy response in HNSCC patients. In this section, we review 69 miRNAs with defined molecular mechanisms, including 25 radioresistant miRNAs and 44 radiosensitive miRNAs. All characteristics are summarized in **Supplementary Table 2**. These miRNAs are involved in mechanisms of radioresistance, which include biological processes, as well as *in vitro* and *in vivo* behaviors. A comprehensive overview of these multifaceted mechanisms—encompassing biological processes (e.g., cell cycle, DNA damage repair), *in vitro* cellular behaviors (e.g., apoptosis, invasion), and *in vivo* tumor growth and metastasis—is schematically depicted in **Figure 2**. The biological processes include cell cycle regulation, DNA damage repair, mitochondrial dysfunction, and DNA methylation. The *in vitro* biological behaviors include cell survival, cell invasion and migration, epithelial-mesenchymal transition (EMT), CSC-like properties, autophagy, apoptosis, and ferroptosis. The *in vivo* biological behaviors include tumor growth and metastasis.

### 3.2 miRNAs in biological processes

#### 3.2.1 miRNAs in cell cycle progression

Cell cycle arrest is the temporary cessation of cell cycle progression at a specific phase, caused by irreparable DNA damage from ionizing radiation (IR) or other insults. Different phases of the cell cycle have different levels of sensitivity to radiation, with the G2/M phase being the most sensitive, whereas the G0 and S phases show relative resistance (22). Notably, numerous recent studies have indicated that miRNAs can exert complex and key regulatory effects on the cell cycle of HNSCC by targeting and regulating cell cycle-related genes and signaling pathways.

Acting as a central mediator in DDR, cell cycle control, and apoptotic pathways, it consequently represents a significant molecular target for cancer therapy. Inhibition of microRNA-17 (miR-17) induces upregulation of the p53 protein, and this elevated p53 then enhances radiation-induced G2/M phase arrest, drives tumor cell apoptosis, and thereby boosts the radiosensitivity of tumor cells (23). Similarly, the downregulation of miR-17 elevates the expression of cyclin-dependent kinase inhibitor 1A (p21). By inhibiting cyclin-dependent kinases, p21 induces G1/S phase arrest. This consequently promotes apoptosis, collectively enhancing the radiosensitivity of HNSCC cells (24). Some studies have also shown that miR-17 expression is upregulated in human radioresistant NPC tissues. By targeting and downregulating *PTEN* expression to activate the PI3K/AKT signaling pathway, miR-17 drives the G1-to-S phase transition of the cell cycle, while also inhibiting radiation-induced apoptosis of tumor cells (25). microRNA-335 can target and inhibit peptidylarginine deiminase 4 expression, thereby negatively regulating the upregulation of p21 expression, leading to cell cycle arrest in the G1 phase and enhanced radioresistance (26). Epstein-Barr virus microRNA-BamHI-A rightward transcript 8 activates the ataxia telangiectasia mutated serine/threonine kinase (ATM)/ataxia telangiectasia mutated serine/threonine kinase (ATR) signaling pathway, thereby enhancing the activities of ATM and ATR, and promoting the phosphorylation of their downstream targets (*e.g*., Checkpoint kinase 1, p53). The activation of these proteins triggers DNA damage-induced cell cycle checkpoints and promotes cell cycle arrest in the G2/M phase (27).

Besides regulating p53- and p21- related cell cycle regulatory proteins, miRNAs can also control the cell cycle of HNSCC cells by regulating other proteins. For example, microRNA-375 inhibits the growth of OSCC cells by targeting insulin-like growth factor 1 receptor, thereby inducing cell cycle arrest in the G0/G1 phase, increasing apoptosis, and enhancing radiosensitivity (28). In general, lncRNAs and circRNAs contribute to radioresistance via miRNA-mRNA axes. Representative molecules include family with sequence similarity 133 member B-2 and long intergenic non-protein coding RNA 00520 (LINC00520), which mediate G1 and G2 phase homeobox A10 (HOXA10) axis cell cycle arrest through the miR-34a/cyclin-dependent kinase 6 axis and microRNA-195, respectively (29, 30).

**Figure 3:**
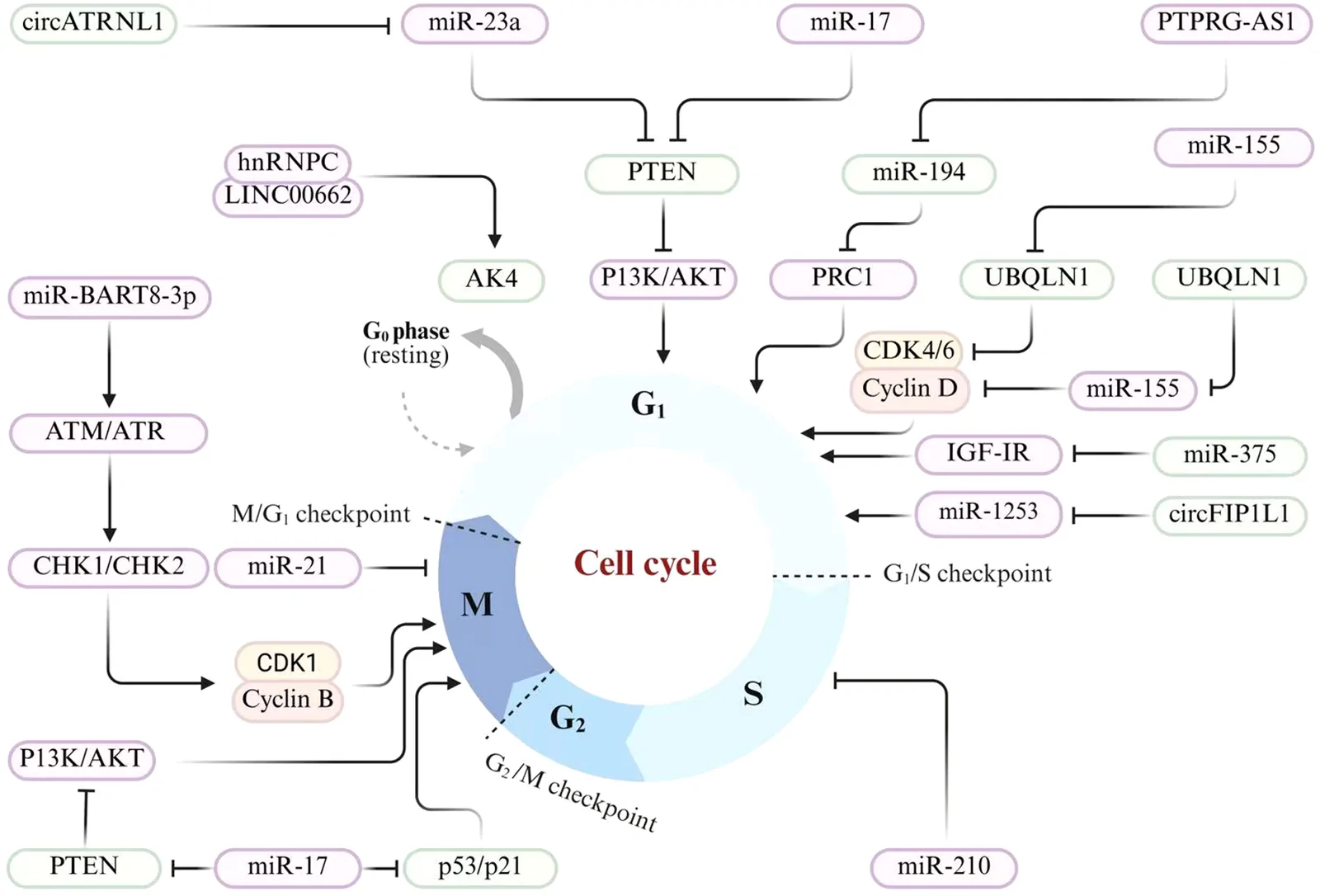
The molecular mechanism by which miRNAs mediate radioresistance through the regulation of the cell cycle. This figure depicts the regulatory networks through which miRNAs, along with lncRNAs and circRNAs, modulate the cell cycle to induce radioresistance. It highlights the cell cycle phases (G_0_, G_1_, S, G_2_, M) and critical checkpoints (G_1_/S, G_2_/M, M/G_1_).

In summary, different miRNAs regulate cell cycle progression and checkpoint activation through multiple targets and pathways in HNSCC, thereby influencing radiosensitivity (**Figure 3**). A deeper understanding of how these miRNAs function paves the way for personalized treatment approaches and the identification of novel targets for radiosensitization in HNSCC.

#### 3.2.2 miRNAs in DNA damage response

The induction of DNA double-strand breaks (DSBs) and subsequent DDR by radiation in HNSCC cells positions these molecules as potent modulators of tumor radiosensitivity (31). As a class of key regulators of the DNA damage response, miRNAs are involved in the regulation of radiosensitivity (32). ATM is an autophosphorylated protein kinase that belongs to the phosphoinositide 3-kinase-related kinase family. Its main function is to sense and respond to DSBs: ATM activates cell cycle checkpoints, DNA repair pathways, apoptosis pathways, and other signaling pathways by phosphorylating downstream targets (*e.g.*, p53, checkpoint kinase 2 (CHK2), phosphorylated histone H_2_AX at Ser-139 (γ-H_2_AX)), thereby protecting cells from loss of genetic information or mutations caused by DNA damage (33). For example, a study showed that Epstein-Barr-virus miR-BamHI promotes DNA repair by activating the ATM/ATR signaling pathway, which in turn reduces DNA damage accumulation and increases radioresistance in NPC cells (27). Another study has shown that miR-421 is overexpressed in HNSCC cells and it targets the 3’-UTR of ATM, resulting in ATM deficiency and leading to DNA repair deficiency in SKC cells. Inhibition of miR-421 can restore the expression of ATM protein, enhance DNA repair capacity, and thus increase radioresistance of HNSCC cells (34).

When a DSB occurs in DNA, ATM/ATR kinase rapidly phosphorylates histone H_2_AX at Ser-139 to form γ-H_2_AX. Subsequently, hundreds of γ-H_2_AX molecules are recruited around each DSB, forming fluorescent foci visible under a microscope. After repair is completed, γ-H_2_AX is dephosphorylated by phosphatases, and the foci gradually disappear (35). DNA damage can therefore be assessed by determining the levels of γ-H_2_AX protein associated with DSBs. For instance, a published study indicated that overexpression of microRNA-483 (miR-483) led to reduced apoptosis and DNA damage caused by irradiation compared to control groups, ultimately decreasing the radiosensitivity of NPC cell lines (36). Huang et al. revealed that microRNA-181a (miR-181a) enhances NPC radioresistance *in vivo* by targeting raf kinase inhibitory protein (RKIP). This was evidenced by γ-H_2_AX staining, which showed attenuated DNA damage in tumors derived from C666–1 NPC cells upon miR-181a overexpression (37). As another radioresistant miRNA, downregulation of miR-21 increases DNA damage, suppresses cell survival and promotes apoptosis; consistently, reduced miR-21 expression also results in decreased tumor size in relevant experimental settings (38).

**Figure 4:**
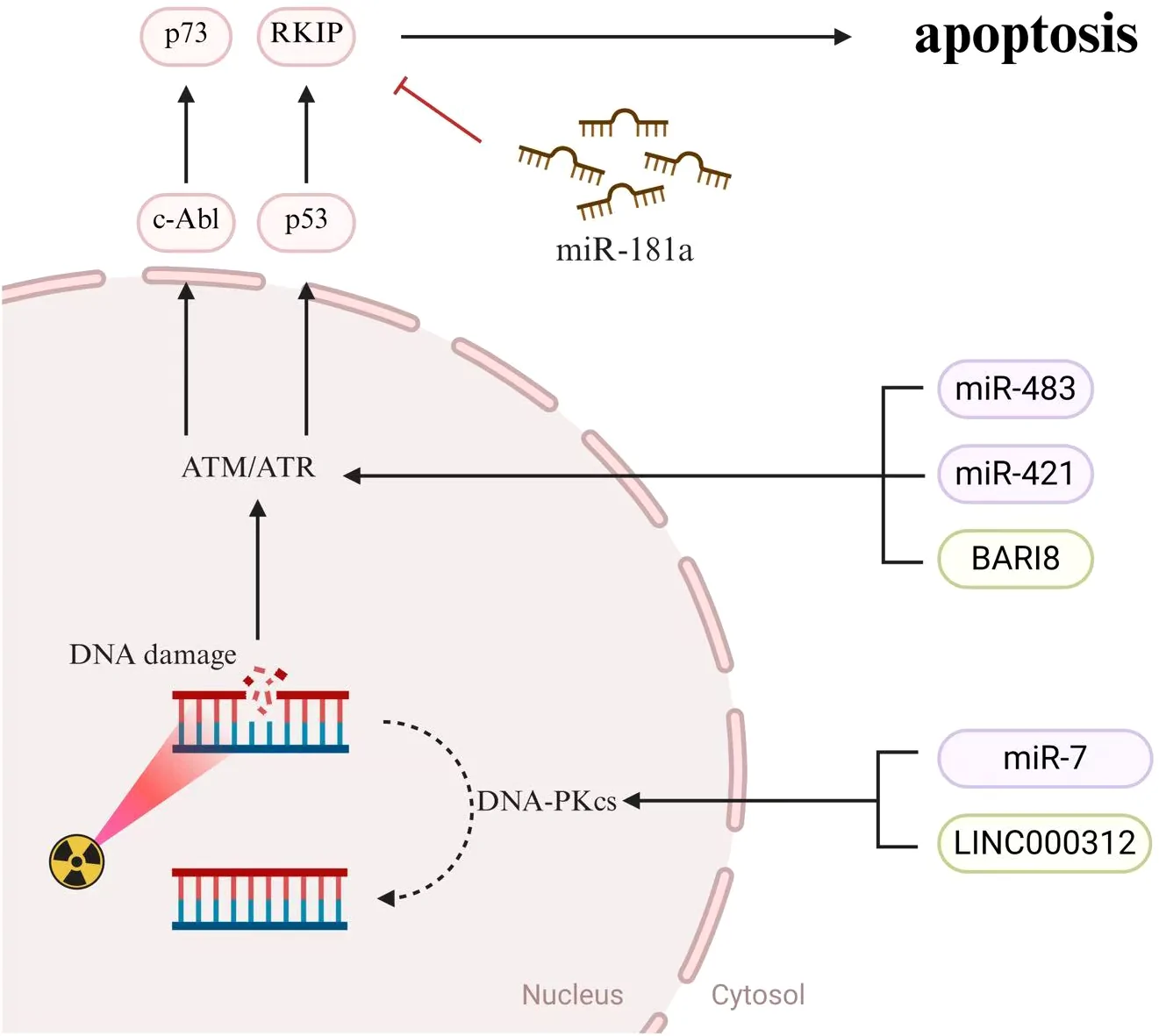
Non-coding RNA regulation of DDR and apoptosis in irradiated cancer cells. This schematic depicts how non-coding RNAs modulate the ATM/ATR and DNA-PKcs DNA damage response pathways.

DNA-dependent protein kinase catalytic subunit (DNA-PKcs) is a key protein in the DNA damage repair pathway and belongs to the phosphoinositide 3-kinase-related kinase family. It is primarily involved in the non-homologous end-joining (NHEJ) pathway and is responsible for repairing DNA DSBs (39). Overexpression of microRNA-7 (miR-7) has been shown to induce the downregulation of DNA-PKcs, reduce DNA damage repair, and promote radioresistance in HNSCC cells (40). In NPC, lncRNAs can directly target DNA-PKcs activity to regulate the DNA repair response after irradiation. For example, long intergenic non-protein coding RNA 00312 (LINC00312) directly binds to DNA-PKcs, impedes the recruitment of DNA-PKcs to adenosine triphosphate (ATP)-dependent DNA helicase 2 subunit 2, inhibits phosphorylation of the AKT-DNA-PKcs axis, consequently blocking DNA damage sensing and signal transduction within the NHEJ repair pathway, thereby enhancing radioresistance (41) (**Figure 4**).

#### 3.2.3 miRNAs in mitochondrial dysfunction and DNA methylation

Metabolic reprogramming is the process by which a cell responds to changes in its internal and external environment by adapting its metabolic pathways to meet specific needs (42). The Warburg effect — a component of metabolic reprogramming that is closely related to mitochondrial dysfunction — describes the phenomenon in which tumor cells preferentially use glycolysis over oxidative phosphorylation for energy generation under aerobic conditions. While this metabolic pattern does not increase the efficiency of ATP production, it enables rapid ATP generation, and leads to lactate accumulation and microenvironment acidification, which reduces local tumor radiosensitivity (43). Notably, recent studies have shown that miRNAs can affect the radioresistance of HNSCC by targeting relevant genes, thereby regulating tumor metabolic reprogramming and leading to mitochondrial dysfunction. For example, microRNA-504 (miR-504) promotes radioresistance in NPC cells by downregulating nuclear respiratory factor 1, which induces metabolic reprogramming and mitochondrial dysfunction. Mechanistically, miR-504 is significantly upregulated in radioresistant cell lines. By negatively regulating nuclear respiratory factor 1 expression and its downstream targets (*e.g*., mitochondrial transcription factor A and oxidative phosphorylation complexes), miR-504 enhances radioresistance, leading to mitochondrial dysfunction, thereby indirectly promoting increased glycolysis (44).

DNA methylation, an important epigenetic modification, impacts tumor cell radioresistance through the regulation of gene expression (45). Studies have found that hypermethylation of the promoter regions of specific genes can lead to gene silencing, which in turn enhances the repair of radiation-induced DNA damage or inhibits apoptotic pathways, resulting in increased tumor cell radioresistance (46). Notably, the role of miRNAs in the context of DNA methylation has been extensively examined in cancer pathogenesis. As evidenced by cellular and *in vivo* studies, microRNA-24 (miR-24) significantly curbs NPC growth through the dual mechanisms of proliferative arrest and apoptosis activation. The expression of miR-24 is regulated by hypermethylation of the promoter region of its precursor (pre-miR-24), and this hypermethylation leads to a decrease in miR-24 expression, which in turn enhances the radioresistance of NPC cells (47).

### 3.3 miRNAs in biological behavior *in vitro*

#### 3.3.1 miRNAs in cancer cell survival

Contemporary studies have indicated that miRNAs play an important role in regulating key biological processes, such as cancer cell viability, proliferation, and senescence, thereby influencing the radioresistance of cancer cells. Research has revealed a multi-level mechanism by which miRNAs target relevant signaling pathways to regulate cancer cell viability. For example, microRNA-526b (miR-526b) regulates cell radiosensitivity in radiotherapy-resistant HNSCC by targeting and suppressing the expression of lysosomal-associated membrane protein 3 (LAMP3). Overexpression of LAMP3 significantly enhances the viability and proliferation of radiation-sensitive cells, whereas knockdown of *LAMP3* inhibits the viability and proliferation of radiation-resistant cells (48). Similarly, miR-29a can target *ADAM12*, reduce the proliferation and viability of OSCC cells, and ultimately enhance their sensitivity to radiotherapy (13). microRNA-626 (miR-626) activates the nuclear factor kappa-light-chain-enhancer of activated B cells signaling pathway by targeting TRAF-interacting protein with forkhead-associated domain family member B, thereby affecting the proliferation and viability of cancer cells and reducing the sensitivity of OSCC to radiotherapy (49). microRNA-206 inhibits the proliferation and viability of NPC cells by targeting insulin-like growth factor 1 and inhibiting the PI3K/AKT pathway, ultimately improving their radiosensitivity (50). The suppression of Annexin A1 (ANXA1) by microRNA-196 enhances cell viability and proliferation, thereby conferring increased resistance to radiation (51). miR-29a enhances radiosensitivity by directly binding to and suppressing collagen type I alpha 1 chain, thereby dampening cell proliferation and viability, which consequently increases the therapeutic efficacy of radiation (52).

miR-483 promotes cancer cell radioresistance by directly targeting and inhibiting the expression of death associated protein kinase 1, suppressing radiation-induced DNA damage, and enhancing cell proliferation (36). microRNA-375 can also target *insulin-like growth factor 1 receptor* and inhibit its expression, thereby regulating cellular processes such as cell cycle progression, ultimately inhibiting tumor cell proliferation and enhancing radiosensitivity (28). microRNA-125b reduces tumor cell proliferation and enhances radiosensitivity by targeting and suppressing *intercellular adhesion molecule 2* expression (53). Bromodomain-containing protein 4 (BRD4), as an important transcriptional regulator in cancer, controls the expression of HPV E6 and E7 oncoproteins and regulates cellular DNA damage repair mechanisms. In HPV-positive HNSCC, overexpressed microRNA-9 (miR-9) can bind to *BRD4* mRNA and negatively regulate the translation of *BRD4*, thereby reducing the transcription levels of HPV E6 and E7, resulting in slower proliferation of cancer cells, decreased cell viability, and enhanced sensitivity to radiotherapy (54). Moreover, miR-9 can also exert its effect of promoting radiosensitivity in NPC by directly targeting platelet-derived growth factor receptor beta expression, thus blocking signaling pathways related to cell proliferation and DNA damage repair (55).

Besides regulating cell viability and proliferation, miRNAs also play a critical role in regulating the cellular senescence phenotype. For instance, microRNA-494 upregulation in OSCC triggers a senescence program, characterized by increased β-galactosidase positivity, elevated cyclin-dependent kinase inhibitor 2A/retinoblastoma protein 1, and suppressed B-cell-specific Moloney murine leukemia virus integration site 1 expression. These changes collectively inhibit proliferation and thereby enhance radiosensitivity (56).

In regulating glycolysis, circ-KIAA0907 functions as an endogenous RNA by sequestering miR-96, thereby derepressing its target unc-13 homolog C (UNC13C) and enhancing its expression. The accumulation of UNC13C significantly reduces glucose uptake and lactic acid production in HNSCC cells, and measurement of the extracellular acidification rate confirms that UNC13C accumulation inhibits glycolysis. Glycolysis is a core energy metabolism pathway in cancer cells, and its inhibition directly impairs the proliferation and survival of HNSCC cells, thereby enhancing radiosensitivity (57).

#### 3.3.2 miRNAs in invasion and migration

miRNAs coordinate metastatic competence by targeting pathways that control cell motility and extracellular matrix (ECM) dismantling. These elements facilitate the multi-stage process of cellular invasion by modulating key pathways, including PI3K/Akt and wingless-type MMTV integration site family (Wnt) signaling, as well as EMT components. This orchestrated regulation enables motile cells to initiate ECM degradation and subsequent tissue penetration.

microRNA-34c (miR-34c) inhibits the Wnt signaling pathway by targeting β-catenin, thereby suppressing malignant behaviors, such as invasion and migration, and enhancing radiosensitivity (58). In NPC cells transfected with miR-34c, overexpression of β-catenin counteracted the inhibitory effect of miR-34c on migration and invasion. microRNA-335 targets p*eptidylarginine deiminase 4* to reduce its regulatory effect on the p21-mechanistic target of rapamycin (mTOR) signaling pathway, thus inhibiting the invasion and migration of NPC cells and enhancing their radiosensitivity (26). The let-7c inhibits cancer stemness and oncogenic activity by targeting and downregulating interleukin-8, thereby reducing cancer cell migration and invasion, effects that are reversed when interleukin-8 is upregulated (59). microRNA-193a impacts cancer cell invasion and migration by directly targeting and downregulating serine/arginine-rich splicing factor 2 expression. Studies have indicated that transfection of microRNA-193a mimics or serine/arginine-rich splicing factor 2 knockdown significantly promotes the migration and invasion abilities of NPC cells, thus lowering their radiosensitivity (60). microRNA-196a directly inhibits the expression of *ANXA1*, and reduced *ANXA1* levels enhance cell migration and invasion, which in turn reduces the radiosensitivity of cancer cells (51). The downregulation of microRNA-218 (miR-218) significantly suppresses cancer cell migration and invasion (61). Conversely, microRNA-182 promotes these metastatic behaviors and enhances radioresistance by inhibiting BCL2 interacting protein 3 (BNIP3), an effect that is rescued by BNIP3 overexpression (62).

In addition, some studies only evaluated the effect of miRNAs on migration. For instance, miR-96 directly suppresses *PTEN*, thereby derepressing the PI3K/Akt signaling pathway. This activation consequently promotes HNSCC cell migration and confers increased resistance to radiotherapy (12). miR-29a directly targets *ADAM12* and inhibits its expression, thereby weakening ADAM12-mediated migration and ultimately reducing migration of OSCC cells (13).

In summary, miRNAs critically coordinate cancer cell invasion, migration, and radiosensitivity. They exert these multifaceted effects by targeting pivotal signaling nodes, thereby exerting multidimensional control over key pathways and processes including Wnt, p21/mTOR, and EMT.

#### 3.3.3 miRNAs in EMT

EMT refers to the process by which epithelial cells lose polarity and transform into a mesenchymal cell phenotype, characterized by the downregulation of epithelial markers (such as E-cadherin) and the upregulation of mesenchymal markers (such as N-cadherin, vimentin) (63). This process plays an important role in embryonic development, tissue repair, and cancer metastasis (64). In tumors, EMT is closely related to invasiveness, metastatic potential, and tumor therapeutic resistance — especially radioresistance (65).

There is a complex regulatory relationship between miRNAs and EMT: miRNAs mainly influence the EMT process by targeting key transcription factors, signaling pathway molecules, and cell adhesion molecules (66). Vimentin and E-cadherin, as classic EMT biomarkers, act as key indicators for the activation of EMT. An earlier study demonstrated that microRNA-185 suppresses the expression of wingless-type MMTV integration site family, member 2B by binding to its 5′ untranslated region (5′UTR). A direct impact on NPC radioresistance was observed upon modulating microRNA-185 or wingless-type MMTV integration site family, member 2B levels, as evidenced by a concomitant shift in EMT biomarkers: vimentin reduction and E-cadherin elevation. These findings posit the regulation of EMT as a mechanism through which these molecules influence treatment response (67). The loss of miR-34c in NPC cells increases the expression of mesenchymal markers but decreases the expression of epithelial markers, suggesting that miR-34c has an anti-EMT effect and thus affects radioresistance (58). microRNA-495 regulates EMT-related markers in NPC cells by targeting glucose-regulated protein 78, thus improving the efficacy of radiotherapy (68). miR-526b can inhibit the expression of *LAMP3*, and downregulation of LAMP3 can reverse the EMT process, thereby enhancing radiosensitivity (48).

These findings suggest that miRNAs can regulate EMT markers and key signaling molecules and pathways through multiple targets, thus providing potential intervention strategies for overcoming radiation resistance in HNSCC.

#### 3.3.4 miRNAs in CSC-like properties

CSCs refer to a special population of cells within tumors that possess self-renewal capabilities and are one of the major factors contributing to radioresistance (69). Cancer stem cell-like cells (CSC-like cells) refer to cells that possess CSC-like properties but do not fully exhibit all the typical characteristics of CSCs (70). Beyond conventional isolation methods, CSC-like cells can be isolated from tumor cells and can also be selected by modulating miRNAs. Compared to the 5.8% proportion of CSC-like cells in the control group, upregulating microRNA-124 (miR-124) reduces this proportion to 2.5%. Based on these findings, we hypothesize that downregulating miR-124 might increase the proportion of CSC-like cells, providing new insights into the screening of CSC-like cells. Increased miR-124 expression inhibits stem-like properties in cancer cells, demonstrated by downregulation of ATP-binding cassette subfamily G member 2, SRY-box transcription factor 2 (SOX2), aldehyde dehydrogenase 1, and POU class 5 homeobox 1 (OCT4), along with decreased sphere formation (71). Similarly, upregulation of microRNA-200 also inhibits sphere formation, survival, and invasion of CSC-like cells, and results in the downregulation of stemness and EMT biomarkers (72). miRNAs can also affect the stemness of not only CSCs but also other tumor cells. For example, upregulating miR-218 inhibits the survival and invasion of CSCs, while downregulating miR-218 promotes sphere formation and invasion of tumor cells (61).

#### 3.3.5 miRNAs in apoptosis

**Figure 5:**
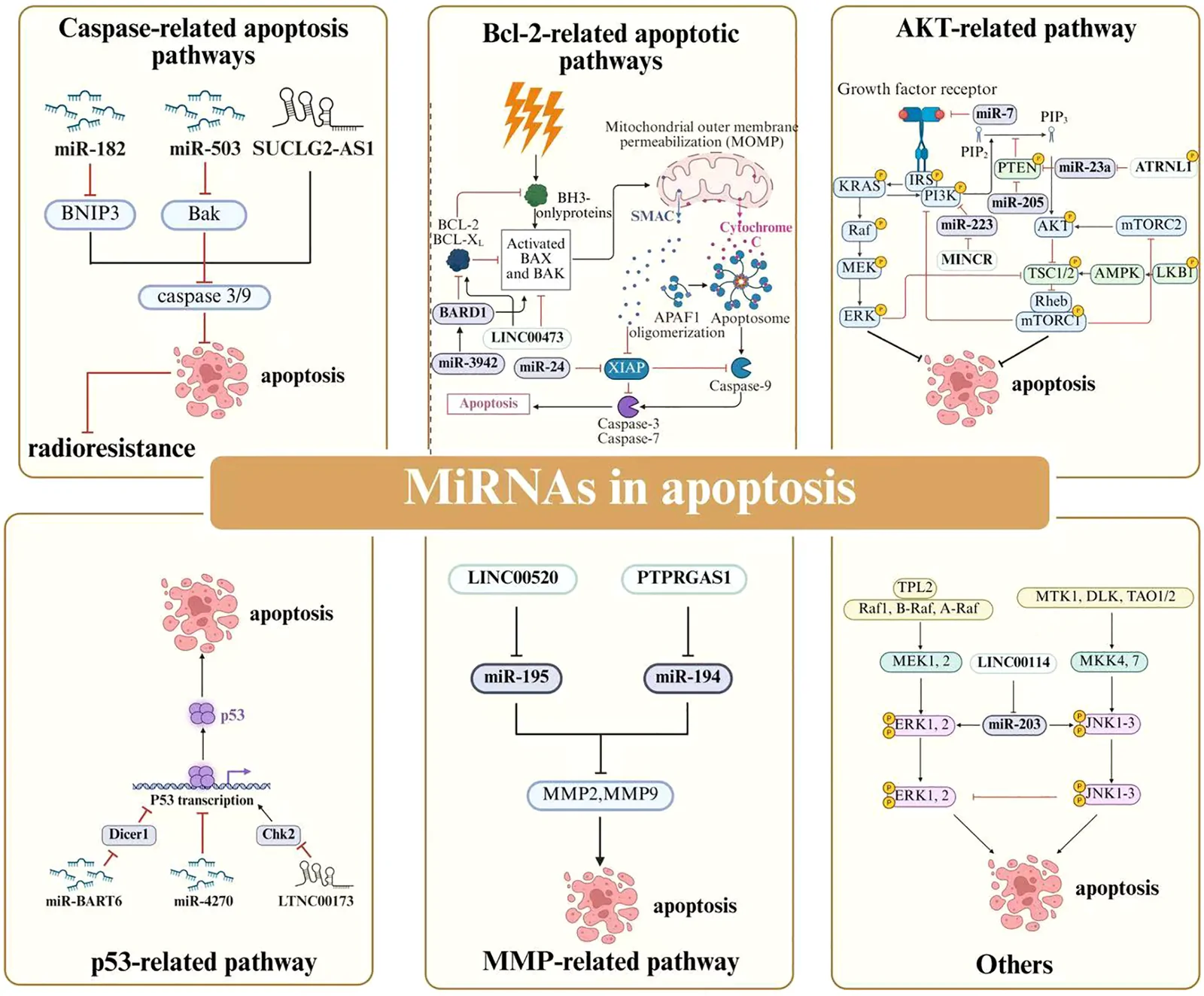
miRNA regulatory networks in apoptosis and cancer radioresistance. This schematic illustrates six key pathways (caspase, Bcl-2, AKT, p53, MMP, and other signaling axes) where miRNAs and associated non-coding RNAs modulate apoptosis to influence cancer radioresistance.

Apoptosis, a type of programmed cell death, is a fundamental and necessary biological process for maintaining morphogenetic homeostasis under early developmental and pathophysiological conditions. Radiation therapy is one of the most important tools in the treatment of head and neck cancers, and it can activate apoptosis in cancer cells by inducing DNA damage and modulating cell cycle progression. Notably, miRNAs function as core regulators of programmed cell death, and via modulating the expression of apoptosis-associated targets, they ultimately enhance radiosensitivity in cancer cells (73). The underlying signaling axes through which miRNAs and other ncRNAs modulate apoptosis—including the caspase, Bcl-2, AKT, p53, MMP, and other pathways—are comprehensively depicted in **Figure 5**.

##### 3.3.5.1 Caspase-related apoptosis pathways

Cysteine aspartate proteases (caspases) are central executors of apoptosis and regulate apoptotic processes through a cascade of activation: initiator caspases (*e.g*., caspase-8/9/10) are activated in response to exogenous or endogenous apoptotic signals, which in turn cleave and activate executor caspases (*e.g*., caspase-3/6/7); the latter induce characteristic apoptotic changes by degrading key substrates (74). miRNAs dynamically regulate the balance between cell survival and apoptosis by directly targeting caspases or their regulatory pathways, and their aberrant expression is closely related to cancer and tumor radioresistance. For example, overexpression of microRNA-182 targets and downregulates *BNIP3* expression, which in turn inhibits the expression of caspase-3 and caspase-9, suppresses apoptosis, and enhances radioresistance (62). Yamana et al. found that microRNA-503 (miR-503) can target and inhibit Bcl-2 homologous antagonist/killer (BAK), disrupting the caspase cascade to suppress radiation-induced apoptosis and enhance radioresistance (75). LncRNAs can also impact tumor radioresistance by directly regulating caspase expression or indirectly regulating it by targeting miRNAs. For instance, Chen et al. found that activation of the long intergenic non-protein coding RNA 662/heterogeneous nuclear ribonucleoprotein C/adenylate kinase 4 axis inhibited the expression of cleaved caspase-3 and enhanced radioresistance (76). Additionally, knockdown of Succinate-CoA ligase GDP-forming beta subunit antisense RNA 1 (SUCLG2-AS1) resulted in increased expression of caspase 3, decreased expression of survivin, and increased apoptosis (77).

##### 3.3.5.2 Bcl-2 family-related apoptotic pathways

The Bcl-2 family is a core protein family that regulates apoptosis in the mitochondrial apoptotic pathway. Its members are divided into anti-apoptotic proteins, pro-apoptotic effector proteins, and BH3-only proteins. BH3-only proteins, upon pro-apoptotic activation, commit the cell to its demise by inducing mitochondrial membrane permeabilization. This is accomplished through the inhibition of anti-apoptotic Bcl-2 members or direct activation of Bcl-2-associated X protein (Bax)/Bak, which triggers cytochrome c release and initiates the caspase cascade. In contrast, anti-apoptotic proteins maintain cell survival by blocking this process. Maintaining the dynamic homeostasis of the Bcl-2 family is essential for development and tissue homeostasis; its dysregulation contributes to abnormal conditions such as apoptosis resistance caused by Bcl-2 overexpression in cancer (78). Recent studies have indicated that miRNAs can directly target Bcl-2 family members to regulate apoptosis. Peng et al. showed in their study that microRNA-3942 (miR-3942) expression was markedly reduced in NPC tissues. Additionally, treatment with a miR-3942 inhibitor resulted in decreased BRCA1 associated RING domain 1 expression, along with reduced expression of the pro-apoptotic protein Bax and increased expression of the anti-apoptotic protein Bcl-2. These findings suggest that miR-3942 exerts dual effects on cells: promoting apoptosis and suppressing tumor cell growth (79). Upregulation of miR-34c reduced the Bcl-2/Bax ratio—a ratio that is negatively correlated with apoptosis levels—suggesting that miR-34c induces apoptosis in NPC cells (58). Expression of lncRNAs can also affect tumor apoptosis and thus tumor radioresistance in HNSCC by directly or indirectly regulating Bcl-2 family expression. Han et al. found that downregulation of long intergenic non-protein coding RNA 00473 (LINC00473) in SCC25 and CAL27 cells significantly increased Bax protein levels and decreased Bcl-2 protein levels. Conversely, overexpression of LINC00473 significantly reduced Bax protein levels and increased Bcl-2 protein levels. These results suggest that LINC00473 can regulate Bcl-2 and Bax expression to impact apoptosis, thereby enhancing HNSCC radioresistance (80).

##### 3.3.5.3 AKT-related apoptotic pathways

The PI3K/AKT signaling pathway suppresses apoptosis primarily by mechanisms involving AKT phosphorylation-mediated inhibition of pro-apoptotic proteins, blockade of caspase activation, and promotion of p53 degradation (81). There is a close bidirectional regulatory relationship between miRNAs and the PI3K/AKT signaling pathway—miRNAs can either control the activity of this pathway via targeting its key proteins or be reversely regulated by the pathway’s downstream effector molecules. In turn, miRNAs contribute significantly to physiological and pathological processes such as cell proliferation, apoptosis, metabolism, and cancer development (82).

Overexpression of miR-205 targets and inhibits *PTEN* expression—*PTEN* acts as a negative regulator of the PI3K/AKT signaling pathway. Inactivation of *PTEN* leads to elevated levels of phosphatidylinositol (3–5)-trisphosphate, which subsequently activates the PI3K/AKT pathway, promoting cell proliferation, inhibiting apoptosis, and enhancing radioresistance (83). miR-17 contributes to radioresistance through its suppression of *PTEN*, which leads to subsequent activation of the PI3K/AKT signaling pathway (25). Circular RNA ATRNL1 (circATRNL1) enhances radiosensitivity by targeting and suppressing microRNA-23a (miR-23a) expression, which alleviates the miR-23a-mediated inhibition of *PTEN* and subsequently inhibits the activation of the PI3K/AKT signaling pathway (84). Overexpression of miR-7 can negatively regulate the epidermal growth factor receptor (EGFR)/PI3K/AKT signaling pathway to enhance radiosensitivity. Therefore, miR-7 holds potential to become a useful therapeutic target for radioresistance caused by the activation of the EGFR/PI3K/AKT signaling pathway (40).

LncRNAs can also modulate the PI3K/AKT signaling pathway by targeting miRNAs; for example, MYC-induced long non-coding RNA (MINCR) targets microRNA-223 to activate the PI3K/AKT pathway, thereby enhancing radioresistance (85).

##### 3.3.5.4 p53-related apoptotic pathways

Radiotherapy induces DNA damage and subsequent apoptosis mediated by the tumor suppressor p53 in cancer treatment. Therefore, targeting the p53-dependent pathway represents a potential strategy to increase apoptosis and improve radiotherapeutic efficacy (86). Downregulation of microRNA-BART6 (miR-BART6) enhances Dicer1 expression, suppresses cancer cell proliferation, invasion, and migration, and promotes apoptosis (87). Notably, Dicer1—acting as a transcriptional co-repressor of p53—is recruited to the promoter regions of p53 downstream target genes through its interaction with p53, playing a role in suppressing p53 transcriptional activity and thus affecting radiation-induced apoptosis (88). Silencing microRNA-4270 enhances tumor-cell sensitivity to irradiation. Mechanistically, microRNA-4270 directly negatively targets *tumor protein p53*, leading to increased p53 expression, which promotes apoptosis and increases radiosensitivity (89). LncRNAs can also regulate tumor radioresistance by modulating p53 expression. For example, in NPC, long intergenic non-protein coding RNA 00173 (LINC00173) reduces apoptosis and enhances radioresistance by inhibiting the Chk2/p53 signaling pathway (90).

##### 3.3.5.5 Matrix metalloproteinase family-related apoptotic pathways

The MMP family is involved in tumorigenesis by bidirectional regulation of apoptosis. On the one hand, certain MMPs (*e.g*., MMP-7 and MMP-3) promote apoptosis by activating death receptors, inducing anoikis, or releasing pro-apoptotic fragments. On the other hand, other MMPs (*e.g*., MMP-9 and MMP-14) inhibit apoptosis by degrading the ECM to activate survival signaling, remove apoptotic ligands, or inhibit caspase activity—ultimately enhancing cell survival (91). Also, lncRNAs/miRNAs regulate apoptosis by modulating MMP expression. For example, silencing LINC00520 in HNSCC cells upregulates microRNA-195 expression, resulting in decreased expression of Bcl-2, MMP-2, and MMP-9, as well as increased expression of Bax, which promotes apoptosis and enhances radiosensitivity (30). Yi et al. found that silencing protein tyrosine phosphatase receptor type G antisense RNA 1 (PTPRG-AS1) upregulates microRNA-194 (miR-194) expression, reduces the expression of MMP-2 and MMP-9, promotes apoptosis, and enhances radiosensitivity in NPC (92).

##### 3.3.5.6 Other regulatory mechanisms

miRNAs can also regulate radioresistance in head and neck tumors through other mechanisms. For instance, the inhibitors-of-apoptosis-protein family—a critical component of these regulatory networks—suppresses apoptosis and sustains cell survival by controlling key death-signaling molecules. Studies from Xu L et al. revealed that miR-24 interacts with the 3’-UTR of X-linked inhibitor of apoptosis protein. Through specific targeting of X-linked inhibitor of apoptosis protein, it inhibits the proliferation of laryngeal squamous cell carcinoma cells, induces apoptosis, and boosts radiosensitivity (93). The mitogen-activated protein kinase family, comprising pivotal regulators of signal transduction, governs essential biological processes—including proliferation, differentiation, stress adaptation, and apoptosis—through specific phosphorylation of downstream substrates. Research has shown that long intergenic non-protein coding RNA 00114 (LINC00114) modulates the extracellular signal-regulated kinase (ERK)/c-Jun N-terminal kinase (JNK) signaling pathway by targeting microRNA-203 (miR-203), which in turn suppresses apoptosis, drives the *in vitro* and *in vivo* tumor progression of NPC, and facilitates radioresistance (94) (**Figure 5**).

#### 3.3.6 miRNAs in autophagy

Certain miRNAs can target autophagy-related proteins to activate tumor-associated autophagic pathways, thereby inducing cell death. The adenosine monophosphate-activated protein kinase (AMPK)/mTOR pathway is critically linked to autophagy regulation and radioresistance: AMPK suppresses mammalian target of rapamycin complex 1 activity by directly phosphorylating mTOR components, thus promoting autophagy and enhancing radioresistance. For instance, microRNA-138 targets eukaryotic translation initiation factor 4E binding protein 1 to promote apoptosis or autophagy and increase radiosensitivity (95). The cancer susceptibility candidate 19 long non-coding RNA (CASC19) relieves the inhibitory effect of microRNA-340 on FK506 binding protein 5 by binding to microRNA-340, thereby releasing FK506 binding protein 5 mRNA, which in turn activates the AMPK/mTOR signaling pathway, inhibits mTOR function, and thus promotes autophagy (96). Another study has shown that si-CASC19 inhibits tumor-cell autophagy by inhibiting the AMPK/mTOR pathway in NPC, and CASC19 has been shown to correlate with radioresistance in NPC (97).

Sequestosome 1 (P62), cytosolic form of LC3 (LC3-I), and lipidated and autophagosome membrane-bound form of LC3 (LC3-II) are critical markers for autophagy, and fluctuations in their expression levels are tightly linked to autophagic activity. Downregulation of microRNA-454 promotes the expression of protein tyrosine phosphatase receptor type D, which directly dephosphorylates signal transducer and activator of transcription 3 to enhance the transcription of autophagy-related 5, which increases the LC3-II/LC3-I ratio, decreases p62 expression, and triggers radiation-induced autophagy (98). miR-526b enhances radiosensitivity in HNSCC by curtailing exosome-mediated LAMP3 transfer. A decreased LC3-II/LC3-I ratio, alongside elevated P62 levels and defective autophagosome formation, collectively points to a substantial attenuation of autophagic flux resulting from this action (48).

#### 3.3.7 miRNAs in ferroptosis

Ferroptosis represents a unique type of regulated cell death. It is driven by iron dyshomeostasis and extensive lipid peroxidation, and is pathologically defined by mitochondrial abnormalities as well as the lethal accumulation of peroxidized polyunsaturated fatty acids (99). Radiation therapy can trigger ferroptosis by inducing the production of reactive oxygen species, thereby enhancing radiosensitivity (100). In NPC, circADARB1 protects cancer cells from radiation-induced oxidative damage and enhances radioresistance by inhibiting ferroptosis (101). A study demonstrated that RRFERV is highly expressed in NPC. Specifically, RRFERV stabilizes TEAD1 expression through competitive binding to miR-615-5p and miR-1293, and exhibits sensitivity to ferroptosis inducers. Collectively, these findings suggest that induction of ferroptosis may serve as a potential therapeutic strategy for reversing radioresistance in NPC (102).

### 3.4 miRNAs in biological behavior *in vivo*

There is growing consensus from recent research that miRNAs are central to the orchestration of tumor-related signaling networks.

Combined overexpression of miR-23a and miR-203 robustly inhibited the growth of NPC tumors, evidenced by diminished tumor weight and volume relative to controls. In addition, a significant increase in the proportion of necrotic areas, a substantial increase in the number of apoptotic cells, and a significant increase in the proportion of γ-H_2_AX-positive cells in tumor tissues were all observed compared with the control group (103, 104). Moreover, microRNA-200c overexpression not only significantly diminished tumor volume and the number of lung metastases *in vivo* but also prolonged the mean survival time of tumor-bearing mice when administered, compared with the control group (72). Overexpression of miR-124 significantly reduced the tumorigenicity of cells, especially at lower cell injection numbers; miR-124 group exhibited lower tumor-forming capacity, slower tumor growth, and lower tumor weight. Furthermore, miR-124 downregulated key stemness-associated proteins (junction adhesion molecule A, OCT4, SOX2), thereby curtailing stem cell-like properties. It also directly enhanced NPC radiosensitivity by targeting the Akt signaling pathway (71). miR-9 significantly slowed tumor growth, and the combination of miR-9 with radiotherapy showed a more significantly reduced tumor growth rate and weight. The miR-9-treated group showed a 60% reduction in tumor volume after radiotherapy and a significantly lower percentage of antigen Ki-67 (Ki-67)-positive cells than the control group (105).

*In vivo* experiments, miR-BART6 significantly affected Dicer1 expression and tumor growth. miR-BART6 inhibitor and radiotherapy combination group had the smallest tumor volume and weight, accompanied by a significant increase in Dicer1 expression—suggesting that miR-BART6 affects the radiotherapy response by regulating Dicer1 (106). In contrast, overexpression of miR-181a enhanced radiotherapy tolerance by inhibiting RKIP, which was manifested by increased xenograft tumor volume and weight, and decreased γ-H_2_AX-positive cells (37). When miR-17 was inhibited in conjunction with radiotherapy, a significant increase in intra-tumoral p53 protein expression was observed. This molecular alteration underlies the observed potent suppression of tumor growth and contributes to markedly enhanced radiosensitivity. Upregulation of p53 expression may further regulate downstream apoptosis-related proteins, thereby enhancing radiotherapy-induced tumor cell death (23). While miR-34c overexpression alone induced only a modest suppression of tumor growth, it exhibited a strong synergistic effect when combined with radiotherapy, potently enhancing the therapeutic outcome. Ki-67 staining showed that miR-34c alone reduced tumor proliferative activity, and this inhibitory effect was further amplified in combination with radiotherapy. In addition, miR-34c alone had a limited ability to induce apoptosis in unirradiated tumors, but significantly increased apoptosis levels after 8 Gy of radiotherapy (58). Without radiotherapy, tumor size in the miR-17 low-expression group was not significantly different from that in the control group. However, in combination with radiotherapy, the size of tumors in the miR-17 knockdown group was significantly reduced after radiotherapy. Analysis of apoptotic cells in tumor sections via the terminal deoxynucleotidyl transferase dUTP nick-end labeling assay revealed a significant increase in cell death within the miR-17 knockdown group compared to the control (24).

Other ncRNAs, including circRNAs and lncRNAs, can also affect radiotherapy sensitivity through unique molecular mechanisms. For example, silencing of the Digeorge syndrome critical region gene 5 (DGCR5) significantly inhibited tumor growth and resulted in a significant decrease in the percentage of Ki-67-positive cells. In addition, Digeorge syndrome critical region 5 silencing upregulated microRNA-506 expression and increased the expression of CSC biomarkers (*e.g*., OCT4, SOX2) (70). Overexpression of circular RNA FIP1-like 1 (circFIP1L1) markedly reduced tumor volume and weight *in vivo*, and these inhibitory effects were further enhanced by 2 Gy radiotherapy. Mechanistically, circFIP1L1 overexpression combined with radiotherapy suppressed cell proliferation (reduced Ki-67-positive staining) and promoted apoptosis. Moreover, circFIP1L1 upregulated PTEN and eukaryotic translation initiation factor 4A3 (EIF4A3) expression, which was augmented by radiation, while downregulating miR-1253. These findings indicate that circFIP1L1 suppresses NPC growth and enhances radiosensitivity via regulating these molecular pathways (107). Tumor growth was significantly restrained by LINC00114 knockdown, and this inhibitory outcome was further strengthened upon combination with a 4 Gy radiation dose. Also, LINC00114 knockdown led to a significant decrease in the phosphorylation levels of key proteins in the ERK/JNK signaling pathway, suggesting that this pathway was inhibited (108). Tumor volume did not change significantly after knockdown of MINCR, but after radiotherapy administration, the MINCR knockdown group showed significantly reduced tumor growth rate and weight, as well as significantly decreased levels of p-AKT and Ki-67 (85). In the lncRNA SUCLG2-AS1 overexpression group, tumor volume and weight were higher than those in the control group, and the percentage of Ki-67-positive cells was increased. In addition, in the popliteal lymph node metastasis model, the SUCLG2-AS1 overexpression group had more metastatic sites than the control group (77). Without radiation, neither overexpression nor knockdown of LINC00173 significantly affected tumor volume relative to controls. However, upon 12 Gy irradiation, tumor volume and weight were significantly increased in the LINC00173 overexpression group but decreased in the knockdown group. TUNEL staining demonstrated that irradiation-induced apoptosis was markedly reduced by LINC00173 overexpression and enhanced by its knockdown (90). Nude mice bearing NPC cells overexpressing LINC00312 exhibited a significant radiosensitizing effect. Specifically, LINC00312 overexpression significantly suppressed tumor growth and reduced tumor weight compared to controls. This inhibitory effect was markedly potentiated by combination with 6 Gy radiotherapy, demonstrating a synergistic interaction (41).

Compared to the multidimensional mechanistic studies mentioned above, some experimental studies on miRNAs focus more on the fundamental regulation of tumor growth, with the main phenotypic changes reflected in tumor volume or weight. Treatment with either 2 Gy radiotherapy or a miR-626 antagonist alone significantly reduced tumor volume and weight, whereas in combination with radiotherapy, a miR-626 antagonist had a stronger inhibitory effect on OSCC tumor growth (49). miR-19b did not affect tumor growth or weight in the non-radiation group, but in combination with radiotherapy, it significantly increased tumor growth and weight compared to the control group (60). NPCs overexpressing miR-17 grew much faster than the control group in terms of volume and weight (25). Overexpression of miR-483 also significantly enhanced tumor cell survival in the context of radiotherapy, leading to significantly larger volume and weight of transplanted tumors after radiotherapy (36). Mice in the circKIAA0907 overexpression group showed significantly reduced tumor volume and weight compared with the control group (57). Additionally, a variety of ncRNAs have been shown to affect tumor volume and weight, including specific miRNAs like microRNA-519d, miR-24, and microRNA-129, in addition to LINC00520 (30, 47, 109–111).

A literature review indicates that miRNAs regulating *in vivo* tumor growth can be classified into radiotherapy-dependent and radiotherapy-independent subtypes. Radiotherapy-independent miRNAs inhibit tumor growth even without radiation, and their suppressive effects are further enhanced by radiotherapy. For instance, miR-9 reduces tumor growth alone and more effectively in combination with radiation; miR-34c mildly inhibits proliferation alone but markedly promotes apoptosis when combined with radiotherapy, suggesting a role in amplifying radiation-induced DNA damage. In contrast, radiotherapy-dependent miRNAs exert regulatory effects only in conjunction with radiation. For example, miR-19b shows no effect on tumor growth without irradiation but promotes tumor progression under radiotherapy. Similarly, miR-17 knockdown does not affect tumor size under basal conditions but significantly reduces tumor volume and weight when combined with radiation. The functions of radiotherapy-dependent miRNAs may rely on radiotherapy-induced microenvironmental changes (e.g., DNA damage, oxidative stress) or synergistic modulation of radiosensitive pathways such as the p53-Bcl-2 axis.

Although the aforementioned studies have systematically elucidated the multi-layered regulatory networks of miRNAs underlying HNSCC radioresistance, it should be noted that nearly all existing evidence is derived from a limited panel of cell lines and xenograft models. These experimental systems fail to adequately recapitulate the profound impacts of the tumor microenvironment (e.g., hypoxia, immune infiltration) and inter-patient heterogeneity on miRNA functionality. Furthermore, the vast majority of investigations adopt a linear “single miRNA–single target” research paradigm, which disregards the crosstalk and functional redundancy among individual miRNAs, as well as between miRNAs and lncRNAs/circRNAs. This limitation may lead to erroneous identification of core regulatory hubs. Future research should leverage cutting-edge technologies including single-cell RNA sequencing and spatial transcriptomics to validate the dynamic characteristics of miRNA regulatory networks in clinically relevant models (such as patient-derived xenografts and tumor organoids) and systematically evaluate the translational potential of these miRNAs as combinatorial therapeutic targets.

## 4 Molecular mechanisms of action of lncRNAs and circRNAs in radioresistance of HNSCC

### 4.1 Overview

LncRNAs are a class of ncRNA molecules with a length of more than 200 nucleotides (112), while circRNAs are closed circular RNA molecules formed by reverse splicing of pre-mRNA. Although neither of them encodes a protein, both can regulate gene expression through various mechanisms, including the regulation of miRNAs (113). In cancer, lncRNAs and circRNAs form a complex gene regulatory network by competitively binding to miRNAs. For example, they can act as “molecular sponges” to sequester miRNAs, thereby blocking the binding of miRNAs to their target mRNAs and relieving the inhibitory effects of miRNAs on their target genes (114, 115). Furthermore, lncRNAs can directly promote or suppress miRNA biogenesis, whereas circRNAs may exert indirect regulatory effects on miRNA function by interacting with RNA-binding proteins (116).

### 4.2 LncRNAs in radioresistance of HNSCC

The regulatory role of lncRNAs has garnered increasing attention in recent years. A total of 22 radioresistant lncRNAs and 4 radiosensitive lncRNAs have been reported. Most studies have shown that lncRNAs promote radioresistance in cancer cells through molecular sponge mechanisms, such as sequestering miRNAs.

LINC00520 acts as a molecular sponge for miR-195, thereby upregulating its target gene HOXA10. Elevated HOXA10 promotes cell proliferation, invasion, and reduces radiosensitivity. LINC00520 silencing or miR-195 overexpression markedly enhanced radiosensitivity in HNSCC cells, as shown by increased apoptosis and reduced proliferation, migration, and invasion following irradiation. In nude mouse xenografts, LINC00520 knockdown suppressed tumor growth and potentiated radiotherapy efficacy *in vivo* (30). LncRNA PTPRG-AS1 acts as a molecular sponge for miR-194, thereby counteracting its repression of polycomb repressive complex 1 (PRC1). This leads to the stabilization of PRC1, a key cytokinesis regulator associated with tumorigenesis. Experimental evidence indicates that either miR-194 overexpression or PTPRG-AS1 silencing disrupts cell cycle progression, resulting in a higher proportion of G1 phase-arrested cells and reduced S phase accumulation. These manipulations concurrently enhance apoptotic activity while suppressing cellular migration, invasion, and proliferative capacity, ultimately enhancing radiosensitivity. In addition, overexpression of miR-194 or inhibition of PTPRG-AS1 can also result in reduced tumor growth and impaired tumor-forming ability in nude mouse xenograft models (92). LncRNA CASC19 mediates radiation resistance in NPC through multiple mechanisms. It induces autophagy through a dual mechanism involving AMPK activation and mTOR suppression, thereby facilitating the clearance of cellular components damaged by radiotherapy. At the same time, CASC19 can also downregulate apoptosis-related molecules such as PARP1 and caspase-3, thereby inhibiting programmed cell death. Experimental studies have demonstrated that knockdown of CASC19 significantly decreased the survival rate of cancer cells and reduced tumor growth rate in nude mouse xenograft models (97). LINC00114 can sequester miR-203, thereby alleviating the inhibitory effect of miR-203 on downstream targets of the ERK/JNK signaling pathway. This leads to increased phosphorylation of downstream targets of the ERK/JNK pathway, ultimately promoting tumor cell proliferation, migration, and invasion, while suppressing radiation-induced apoptosis. Cellular and animal studies consistently demonstrated that LINC00114 knockdown suppresses NPC tumorigenicity by inhibiting colony formation, inducing apoptosis, and promoting radiosensitization (108). Besides the molecular sponge mechanism, some lncRNAs promote radioresistance through protein binding and signaling axis activation. For example, RORA-AS1 promotes radiotherapy resistance in OSCC cells by binding to IFITM1 and activating the IFITM1/STAT/p21 signaling axis. Both *in vitro* and *in vivo* experiments have confirmed that knockdown of RORA-AS1 attenuates radiotherapy resistance induced by CAF-derived exosomes, suggesting that RORA-AS1 may serve as a potential therapeutic target to improve the efficacy of radiotherapy for OSCC (117).

However, certain studies have revealed that a few lncRNAs have the opposite function, that is, they enhance radiosensitivity by regulating key signaling pathways. For instance, by sponging microRNA-452, long intergenic non-protein coding RNA 01140 attenuates microRNA-452-mediated inhibition of zinc finger protein 621 and upregulates zinc finger protein 621 expression, thereby suppressing DNA repair and enhancing radiation-induced apoptosis, ultimately enhancing the radiosensitivity of NPC cells (94). LINC00312 directly binds to DNA-PKcs, blocks its recruitment to adenosine triphosphate (ATP)-dependent DNA helicase 2 subunit 2, and inhibits AKT-DNA-PKcs phosphorylation, thereby impairing DNA damage sensing and signaling during NHEJ repair. Furthermore, LINC00312 suppresses both the Mre11-Rad50-Nbs1/ATM/CHK2 and ATR/checkpoint kinase 1 pathways, leading to inhibited DNA repair and dysregulated cell cycle progression. Accumulated DNA damage downregulates anti-apoptotic proteins such as Bcl-2, ultimately enhancing radiation-induced apoptosis and reversing radioresistance (41). Moreover, Long non-coding RNA Family with Sequence Similarity 133 Member B transcript variant 2 can inhibit the radioresistance of NPC cells and affect their proliferation and apoptosis. It also targets microRNA-34a and regulates cyclin-dependent kinase 6 (29).

These mechanisms collectively reveal the critical role of lncRNAs in cancer and establish a theoretical foundation for developing novel biomarkers and therapeutic targets.

### 4.3 CircRNAs in radioresistance of HNSCC

Accumulating evidence indicates that circRNAs can orchestrate cancer cell radiosensitivity by modulating pivotal biological processes—including DNA damage repair, apoptosis, and ferroptosis—along with key signaling pathways. At least 7 radioresistant circRNAs and 3 radiosensitive circRNAs have been reported to date.

CircRNA_000543 is upregulated in NPC tissues, and high expression correlates with poorer overall survival (OS). Knockdown of circRNA_000543 suppresses proliferation and invasion, and induces apoptosis in NPC cells. In xenograft models, silencing circRNA_000543 reduces tumor volume and enhances radiosensitivity in human nasopharyngeal carcinoma cell line 1 radioresistant cells. Mechanistically, circRNA_000543 acts as a sponge for miR-9, thereby upregulating platelet-derived growth factor receptor beta, promoting cell survival and invasion, and increasing radioresistance (55). Also, circADARB1 upregulates heat shock protein 90 beta family member 1 to stabilize solute carrier family 7 member 11 and glutathione peroxidase 4, maintain glutathione homeostasis, and inhibit lipid peroxidation, thereby suppressing ferroptosis and conferring radioresistance (101). Additionally, circular RNA CDYL2 can function as a molecular scaffold to recruit Eukaryotic translation initiation factor 3 subunit D protein for binding to RAD51 recombinase (RAD51) and promote its translation initiation, thereby increasing RAD51 protein levels. This process enhances homologous recombination repair capacity, enabling tumor cells to repair radiation-induced DNA damage more efficiently and ultimately resulting in radioresistance (118). Circular RNA CUX1 (CircCUX1) enhances its own stability through N6-methyladenosine modification, suppresses caspase 1 expression, and inhibits the secretion of downstream inflammatory factors. Experimental evidence demonstrates that circCUX1 knockdown significantly reduces cell viability and increases the release of inflammatory factors Interleukin-1 beta and Interleukin-18 (119).

Conversely, circFIP1L1 overexpression combined with miR-1253 knockdown suppresses NPC cell proliferation, induces apoptosis, and enhances radiosensitivity. Mechanistically, EIF4A3 binds to FIP1L1 mRNA and facilitates its circularization into circFIP1L1. Acting as a competing endogenous RNA, circFIP1L1 sponges miR-1253, relieving its inhibition on EIF4A3 and PTEN. Meanwhile, EIF4A3 directly binds and stabilizes PTEN mRNA, synergistically upregulating PTEN. PTEN, a critical tumor suppressor, inhibits proliferation, promotes apoptosis, and reverses radioresistance by inactivating the PI3K/AKT pathway (107). Moreover, the aforementioned circKIAA0907 alleviates the inhibitory effect on its target gene *UNC13C* by specifically sponging miR-96-5p, thereby upregulating *UNC13C* expression. The circKIAA0907-induced accumulation of UNC13C protein significantly reduces the glucose uptake capacity of cancer cells, decreases lactic acid production, and impairs glycolysis, thereby suppressing their proliferative capacity and enhancing their sensitivity to radiotherapy (57). CircATRNL1 alleviates the suppression of *PTEN* by sponging miR-23a-3p, thus suppressing the activity of the PI3K/AKT pathway. Experimental studies have demonstrated that the upregulation of circATRNL1 suppresses the proliferation of OSCC cells, induces apoptosis and cell cycle arrest, and ultimately enhances the radiosensitivity of OSCC cells (84).

In summary, circRNAs markedly influence radioresistance in NPC by regulating key molecular pathways. Preclinical studies validate that circRNAs like circRNA_000543 and circFIP1L1 are pivotal for modulating radioresistance, solidifying their dual role as therapeutic vulnerabilities and biomarkers. Compared with miRNAs, research on lncRNAs and circRNAs is still in its infancy. Most available publications focus on the ceRNA mechanism, whereas few studies have explored their miRNA-independent regulatory functions, such as chromatin remodeling, transcriptional regulation, and protein scaffolding. Notably, the circular conformation of circRNAs endows them with superior stability, yet it poses technical obstacles to their detection and quantification. Discrepancies in qPCR primer design and RNase R digestion conditions across independent studies may render experimental results incomparable. Moreover, lncRNAs and circRNAs exhibit prominent tissue specificity, and systematic comparisons of their expression profiles and functional discrepancies across distinct HNSCC subtypes are scarce. Accordingly, it is urgent to systematically dissect the non-redundant functions and clinical relevance of these molecules via CRISPR screening and high-throughput functional genomics within a standardized experimental framework.

## 5 Clinical significance of ncRNAs for HNSCC and NPC patients after radiotherapy

### 5.1 Overview of reported potential ncRNAs with clinical significance

**Figure 6:**
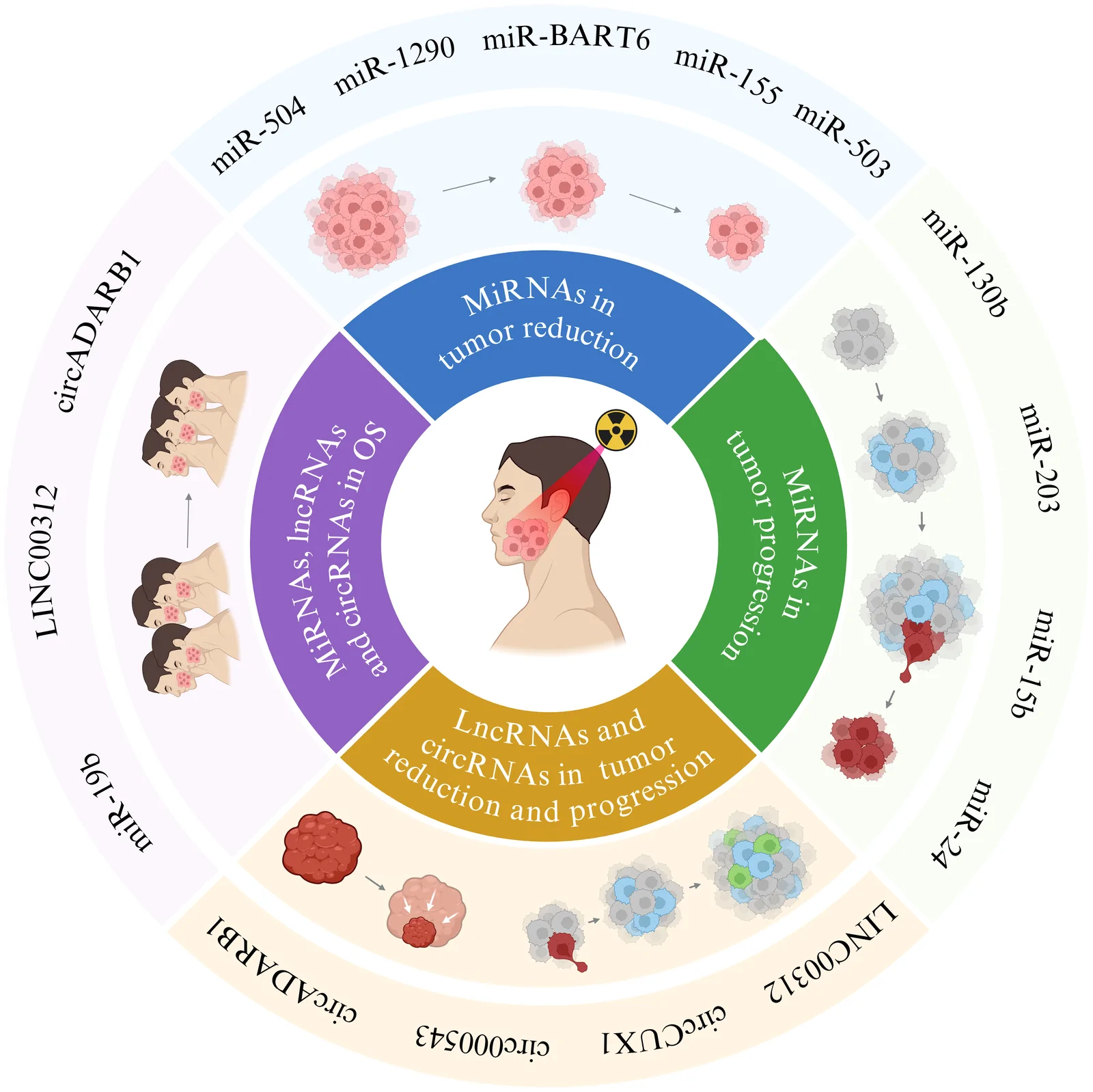
Clinical significance of ncRNAs for HNSCC and NPC patients after radiotherapy. This annular diagram illustrates the functional roles of miRNAs, lncRNAs, and circRNAs in modulating tumor reduction, progression, OS, and radiosensitivity in nasopharyngeal carcinoma during radiotherapy.

To promote the clinical application of ncRNAs in distinguishing between radiosensitive and radioresistant HNSCC patients, we have summarized ncRNAs that have been shown to be related to the radiotherapy prognosis of HNSCC. Furthermore, while radiotherapy is the primary treatment for NPC patients, few studies on NPC, unlike those on HNSCC, specify whether the enrolled patients are classified as having squamous cell carcinoma. Considering that a portion of NPC cases are also squamous cell carcinoma, this lack of clarity regarding squamous cell carcinoma classification in NPC studies has significant implications for research on HNSCC. Therefore, we have included these studies in our review, even if the authors did not specify whether the enrolled clinical NPC patients were diagnosed with squamous cell carcinoma, provided that their preclinical experiments used nasopharyngeal squamous cell carcinoma cell lines. These ncRNAs and their potential clinical applications are summarized in **Supplementary Table 3**. Our review of these studies reveals that clinicians often differentiate radioresistant from radiosensitive patients based on three prognostic outcomes: tumor regression, tumor progression, and OS (**Figure 6**).

A total of 55 ncRNAs have been reported to have the potential to distinguish between radiosensitive and radioresistant enrolled HNSCC or NPC patients, including 18 radiosensitive miRNAs, 21 radioresistant miRNAs, 9 radioresistant lncRNAs, 2 radiosensitive lncRNAs, 4 radioresistant circRNAs, and 1 radiosensitive circRNA. Notably, miR-23a (103, 120) and miR-203 (104, 121) have each been reported in two independent studies as radiosensitive miRNAs, while miR-150 (122, 123) has also been reported in two independent studies as a radioresistant miRNA. These corroborating results suggest that these miRNAs have greater potential for clinical application.

### 5.2 miRNAs in tumor reduction

The degree of tumor reduction after radiotherapy is a widely used indicator in clinical practice. By monitoring changes in tumor volume at different time points after treatment in NPC patients, it has been found that at multiple time intervals (*e.g*., 1 month, 3 months post-radiotherapy), patients with low serum miR-504 have a smaller percentage of remaining tumor volume compared to those with high serum miR-504. Therefore, serum miR-504 holds promise as a time-dependent biomarker to predict the radiotherapy prognosis of NPC patients (44).

Treatment response refers to the clinical condition of tumors after radiotherapy, including complete response (CR), partial response (PR), stable disease (SD), and progressive disease (PD). These four indicators are defined based on changes in tumor volume (increase or decrease) and the presence of new lesions after radiotherapy. Based on therapeutic outcomes, an operational classification defines CR/PR as radiosensitive and SD/PD as radioresistant. For example, the expression level of miRNA-155 is higher in the radioresistant group of NPC patients than in the radiosensitive group (124). Similarly, miR-BART6 is also more highly expressed in the radioresistant group of NPC patients. Its favorable area under the receiver operating characteristic (ROC) curve value of 0.757 (indicating moderate-to-high diagnostic accuracy) demonstrates a strong ability to distinguish between radiosensitive and radioresistant NPC patients (106).

Histological response is a grading standard proposed by Shimosato et al., which is based on the use of histological techniques to monitor the post-radiotherapy tumor tissue status. The response is classified into Grades I-IV based on the degree of tumor tissue destruction, tumor cell activity, and the persistence of tumor cells. Compared to traditional grading systems, this standard provides guidance for precision therapy from a pathological perspective. For example, low expression of miRNA-1290 is more prevalent in HNSCC patients with pathological response Grade ≤ IIa (125). Similarly, high expression of serum miR-503 is more common in HNSCC patients with a pathological response of Grade ≤ IIa. Given that blood tests are routine clinical examinations, predicting patient radiosensitivity based on serum miR-503 levels has promising clinical application potential (75).

### 5.3 miRNAs in tumor progression

Tumor progression after radiotherapy is a significant challenge in the treatment of patients with HNSCC and NPC. Therefore, identifying biomarkers associated with tumor progression has important clinical significance. The expression level of miR-24 in recurrent NPC tissues is approximately 50% lower than that in primary NPC tissues, suggesting that miR-24 may serve as a potential biomarker for recurrence (47). Recurrence is a time-dependent indicator; thus, comparing miRNA expression differences between primary and recurrent tumor tissues is less clinically meaningful than comparing such differences among patients with different recurrence times (or no recurrence). For example, the expression level of miR-15b is lower in HNSCC patients with short-term locoregional control compared to those with long-term locoregional control. ROC curve analysis further demonstrated that miR-15b can distinguish between HNSCC patients with long-term and short-term locoregional control, with an area under the ROC curve of 0.719. Local relapse-free survival analysis provided additional support for this finding (126).

In addition to relapse-free (or recurrence-free) survival, disease-free survival (DFS) and progression-free survival (PFS) are also commonly used clinical indicators to evaluate disease progression. For instance, HNSCC patients with high miR-130b expression have poorer PFS, while NPC patients with low miR-203 expression have poorer DFS (104, 127).

As mentioned earlier, treatment response also reflects tumor progression to some extent, as it includes the category of PD—which includes the presence of new lesions (124). A similar treatment response indicator is frequently used in NPC studies. This indicator is time-dependent and focuses on the duration of tumor regression or the occurrence of recurrence. Different researchers use different observation time points, with common ones including 6 weeks, 2 months, 3 months, and 12 months (58, 68, 103).

### 5.4 LncRNAs and circRNAs in tumor reduction and progression

The expression of LINC00312 can distinguish patients with different treatment responses (*e.g*., CR *vs*. PD) (41), circCUX1 can differentiate patients with different DFS outcomes (*e.g*., shorter *vs*. longer DFS) (116); circRNA_000543 can identify patients with different outcomes of tumor regression or local recurrence (55), and circADARB1 or circular RNA CDYL2 can distinguish patients with recurrence or metastasis within five years from those without recurrence or metastasis within the same five-year period (101, 118).

### 5.5 miRNAs, lncRNAs and circRNAs in OS

OS refers to the time from the start of a particular treatment regimen to death from any cause. It is considered the primary efficacy endpoint in cancer clinical trials (128). Three types of ncRNAs have been reported to be associated with OS, including miR-19b, LINC00312, and circADARB1 (41, 101, 129). Additionally, it is worth noting that some researchers have focused on the combined use of two miRNAs to assess their association with OS, rather than relying on a single miRNA for this purpose. This combined approach may offer a higher or unique prognostic value (130).

## 6 Conclusion

Radiotherapy plays a crucial role in the management of advanced or inoperable cases of HNSCC, but its efficacy is limited by the radioresistance of HNSCC. ncRNAs, including miRNAs, lncRNAs, and circRNAs, have emerged as key regulators of gene expression, influencing various cellular processes involved in tumor progression and radioresistance in HNSCC.

The findings summarized in this review underscore the importance of ncRNAs in modulating the radiosensitivity of HNSCC, highlighting both their potential as biomarkers for predicting treatment outcomes and their role in enhancing radiotherapeutic efficacy. While much attention has been given to miRNAs, the roles of lncRNAs and circRNAs remain underexplored but represent promising avenues for future research. A deeper understanding of these molecules could lead to the development of novel therapeutic strategies to overcome radioresistance in HNSCC.

In conclusion, targeting ncRNAs represents a novel therapeutic dimension for HNSCC, holding significant potential to enhance radiotherapy efficacy and ultimately improve patient prognosis. Although this review systematically summarizes numerous important advances, several major limitations should be noted. A substantial gap persists in clinical translation of preclinical findings; most available evidence originates from *in vitro* cell-culture assays and *in vivo* xenograft models, which cannot faithfully recapitulate the inherent complexity of the HNSCC tumor microenvironment and its genetic heterogeneity (131). Furthermore, prevailing studies largely focus on single ncRNA molecules, whereas insufficient efforts have been devoted to deciphering the complex regulatory networks and functional redundancy across miRNAs, lncRNAs, and circRNAs (132). For example, one miRNA can target hundreds of transcripts concurrently, and a single lncRNA may sponge multiple miRNAs; such many-to-many interactions indicate that a systems-biology framework is required to dissect core network nodes and prioritize optimal therapeutic targets (133). In addition, ncRNA-based biomarkers are still lacking rigorous validation in large-scale, independent prospective cohort studies, and their clinical translational value remains to be fully confirmed.

## Supplementary material

Supplementary Table 1 Summary of articles screening and inclusion. This table summarizes the outcomes of systematic literature retrieval and study screening. A total of 345 literatures were screened.

Supplementary Table 2 Summary of molecular mechanisms of ncRNA involved in radioresistance and radiosensitivity. A data table of RNA radiation sensitivity/resistance characteristics, focusing on the biological characteristics of RNA molecules in radiation related research.

Supplementary Table 3 Summary of clinical correlation and prognostic value of ncRNA in radiotherapy. This table is a prognostic association data table for RNA radiotherapy, focusing on the association between RNA molecules and patient prognosis outcomes after radiation therapy.

## Glossary

ADAM12: ADAM metallopeptidase domain 12
AMPK: Adenosine monophosphate-activated protein kinase
ANXA1: Annexin A1
ATM: Ataxia telangiectasia mutated serine/threonine kinase
ATP: Adenosine Triphosphate
ATR: Ataxia telangiectasia and Rad3 related checkpoint kinase
Bak: Bcl-2 homologous antagonist/killer
Bax: Bcl-2-associated X protein
Bcl-2: B-cell lymphoma 2
BNIP3: BCL2 Interacting Protein 3
BRD4: Bromodomain-containing protein 4
CASC19: Cancer susceptibility candidate 19 long non-coding RNA
Caspase: Cysteine aspartate proteases
Chk2: Checkpoint kinase 2
circATRNL1: Circular RNA ATRNL1
circCUX1: Circular RNA CUX1
circFIP1L1: Circular RNA FIP1L1
circKIAA0907: Circular RNA KIAA0907
circRNAs: Circular RNAs
CR: Complete response
CSCs: Cancer stem cells
DDR: DNA damage response
DFS: Disease-free survival
DNA-PKcs: DNA-dependent protein kinase catalytic subunit
DSBs: DNA double-strand breaks
ECM: Extracellular matrix
EIF4A3: Eukaryotic translation initiation factor 4A3
EMT: Epithelial-mesenchymal transition
ERK: Extracellular signal-regulated kinase
FIP1L1: FIP1-like 1
HNSCC: Head and neck squamous cell carcinomas
HOXA10: Homeobox A10
HPV: Human papillomavirus
IGF-1R: Insulin-like growth factor 1 receptor
IR: ionizing radiation
JNK: c-Jun N-terminal kinase
Ki-67: Antigen Kiel-67
LAMP3: Lysosomal-associated membrane protein 3
LC3: Microtubule-associated proteins 1A/1B light chain 3B
LC3-I: Cytosolic form of LC3
LC3-II: Lipidated and autophagosome membrane-bound form of LC3
LINC00114: Long intergenic non-protein coding RNA 00114
LINC00173: Long intergenic non-protein coding RNA 00173
LINC00312: Long intergenic non-protein coding RNA 00312
LINC00473: Long intergenic non-protein coding RNA 00473
LINC00520: Long intergenic non-protein coding RNA 00520
lncRNAs: Long non-coding RNAs
LSCC: Laryngeal squamous cell carcinoma
MINCR: MYC-Induced Long Non-Coding RNA
miR-124: microRNA-124
miR-17: microRNA-17
miR-181a: microRNA-181a
miR-194: microRNA-194
miR-203: microRNA-203
miR-218: microRNA-218
miR-23a: microRNA-23a
miR-24: microRNA-24
miR-34c: microRNA-34c
miR-3942: microRNA-3942
miR-483: microRNA-483
miR-503: microRNA-503
miR-504: microRNA-504
miR-526b: microRNA-526b
miR-626: microRNA-626
miR-7: microRNA-7
miR-9: microRNA-9
miR-96: microRNA-96
miR-BART6: microRNA-BART6
miRNAs: microRNAs
MMP: Matrix metalloproteinase
MMPs: Matrix Metalloproteinases
mTOR: Mammalian target of rapamycin
ncRNAs: Non-coding RNAs
NHEJ: Non-homologous end-joining
NPC: Nasopharyngeal carcinoma
OCT4: POU class 5 homeobox 1
OS: Overall survival
OSCC: Oral squamous cell carcinoma
P62: Sequestosome 1
PD: Progressive disease
PI3K/Akt: Phosphatidylinositol 3-kinase/Protein kinase B
PIKK: phosphoinositide 3-kinase-related kinase
PR: Partial response
PFS: progression-free survival
PTEN: Phosphatase and tensin homolog deleted on chromosome ten
PTPRG-AS1: Protein tyrosine phosphatase receptor type G antisense RNA 1
RAD51: RAD51 recombinase
ROC: Receiver operating characteristic
SD: Stable disease
SOX2: SRY-box transcription factor 2
SUCLG2-AS1: Succinate-CoA ligase GDP-forming beta subunit antisense RNA 1
UNC13C: Unc-13 Homolog C
Wnt: Wingless-type MMTV integration site family
γ-H_2_AX: Phosphorylated histone H_2_AX at Ser-139
